# Vitamin D Analogs Potentiate the Antitumor Effect of Imatinib Mesylate in a Human A549 Lung Tumor Model

**DOI:** 10.3390/ijms161126016

**Published:** 2015-11-13

**Authors:** Ewa Maj, Beata Filip-Psurska, Marta Świtalska, Andrzej Kutner, Joanna Wietrzyk

**Affiliations:** 1 Ludwik Hirszfeld Institute of Immunology and Experimental Therapy, Polish Academy of Sciences, Rudolfa Weigla 12, 53-114 Wroclaw, Poland; ewa.maj@iitd.pan.wroc.pl (E.M.); fillip@iitd.pan.wroc.pl (B.F.-P.); switalska@iitd.pan.wroc.pl (M.S.); 2 Pharmaceutical Research Institute, Rydygiera 8, 01-793 Warsaw, Poland; a.kutner@ifarm.eu

**Keywords:** NSCLC, imatinib, vitamin D analogs

## Abstract

In previous papers, we presented data on studies on the anticancer activity of the vitamin D_3_ analogs, named PRI-2191 and PRI-2205, in different cancer models. In this study, we showed the improved antiproliferative activity of a combination of imatinib mesylate (Gleevec, GV) and cytostatic agents in *in vitro* studies, when used with a third compound, namely PRI-2191, in an A549 human lung cancer model. Furthermore, we analyzed the influence of both PRI-2191, as well as PRI-2205 on the anticancer activity of GV in mice bearing A549 tumors. The route of PRI-2191 analog administration showed a significant impact on the outcome of GV treatment: subcutaneous injection was more efficient and less toxic than oral gavage. Moreover, both vitamin D compounds increased the anticancer activity of GV; however, they might also potentiate some adverse effects. We also evaluated in tumor tissue the expression of VEGF, PDGF-BB, vitamin D receptor, CYP27B1, CYP24, p53 and Bcl-2, as well as PDGF receptors: α and β. We observed the upregulation of p53 expression and the downregulation of Bcl-2, as well as VEGF in A549 tumors as a result of the tested treatment. However, vitamin D analogs did not significantly influence the expression of these proteins.

## 1. Introduction

Imatinib mesylate (Gleevec, GV) selectively inhibits the activity of Bcr/Abl, c-Kit and PDGFR kinases [1]. It reveals distinct and rapid antileukemic activity in chronic myelogenous leukemia (CML) and Philadelphia-positive (Ph+) acute lymphoblastic leukemia (ALL) [2]. Moreover, imatinib inhibited c-Kit tyrosine kinase phosphorylation and *in vitro* kinase activity in small cell lung cancer (SCLC) cells [3]. It also inhibited the proliferation and tumor growth of the human A549 non-small-cell lung cancer (NSCLC) model due to the inhibition of PDGFR-β phosphorylation and downregulation of tumor VEGF expression [4,5]. Additionally, there are studies analyzing the use of imatinib in combination with other anticancer agents. For example, Skorta *et al.* showed that imatinib is able to sensitize cells expressing Bcr-Abl fusion protein to cisplatin via interfering with p53 transactivation, elicitation of p53 accumulation mainly in the cytoplasm and decreasing Bcl-xL [6]. Furthermore, the combination of imatinib and cisplatin is sufficiently tolerated and may assure stabilization in locally-advanced and metastatic adenoid cystic salivary gland carcinoma [7]. In a pilot study, involving patients with CML in the blastic phase, imatinib was shown to also act synergistically with idarubicin: the hematologic response was achieved in 74% of patients, among which 47% had complete hematologic remission and 26% returned back to the chronic phase [8]. In *in vitro* studies, it was shown that idarubicin in combination with imatinib influenced the distribution of HL-60 in different phases of the cell cycle, e.g., an increase in G_2_/M phase cell accumulation with a parallel decrease in the G_0_/G_1_ phase was observed [9]. Such findings suggested that therapeutics that can improve the activity of imatinib mesylate may be required to improve this activity and overcome resistance and relapse. A number of preclinical and clinical studies of imatinib mesylate combined with other standard anticancer agents were conducted in order to improve response rates and prolong the survival of the patients [5,10,11,12].

The hormonally-active form of vitamin D_3_ (calcitriol, 1,25-(OH)2D_3_) or its analogs reveal anticancer activity, both *in vitro*, as well as *in vivo*. Calcitriol is engaged in regulating the proliferation and differentiation of a number of normal and cancer cells, including lung cancer cells [13,14,15,16,17,18]. For example, nuclear vitamin D receptor (VDR) status was found to be a prognostic marker in NSCLC [19]. Moreover, the studies with VDR knock-out mice showed that the absence of VDR in tumor-infiltrating vessels resulted in an increased content of angiogenic factors, such as HIF-1α, VEGF, Ang1 and PDGF-BB, in tumors [20]. There are also results showing that there are intermediates of vitamin D_3_ metabolism within the body that are biologically active, showing antiproliferative activity [21,22].

**Figure 1:**
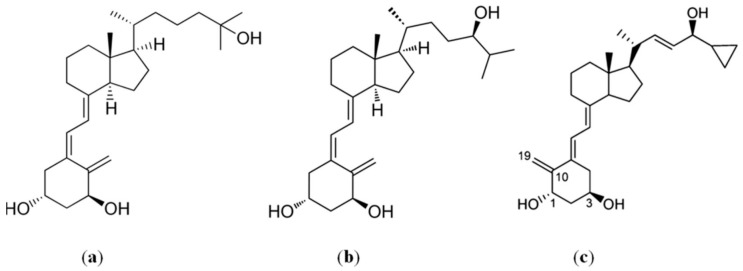
Chemical structure of (**a**) calcitriol, (**b**) PRI-2191 and (**c**) PRI-2205.

Numerous vitamin D_3_ analogs were synthesized in the last two decades in order to obtain agents with advantageous biological and therapeutic properties [13,14,15,23,24,25,26,27]. Previously, we examined vitamin D_2_ analogs having a highly unsaturated side-chain and vitamin D_3_ analogs with one or two additional hydroxyl groups in the side-chain, as well as geometric and diastereomeric analogs of calcipotriol, to assess their *in vitro* and *in vivo* activity against various human normal and cancer cell lines. Based on these studies, we selected the active metabolite PRI-2191 ((24R)-1,24-dihydroxyvitamin D_3_, tacalcitol) and analog PRI-2205 (5,6-trans-calcipotriol) (PRI = **P**harmaceutical **R**esearch **I**nstitute) as the most potent and the least toxic vitamin D compounds (Figure 1) [14,15,25,28,29,30].

The vitamin D compounds were demonstrated as interesting candidates for combined anticancer treatment, especially with cytostatic drugs [31,32,33]. Moreover, our and others’ previous studies revealed that both calcitriol and its analogs improved the antiproliferative activity of imatinib towards leukemia, mastocytoma and head and neck cancer cell lines [9,34,35].

In this paper, we presented the cytotoxic activity of imatinib mesylate in conjunction with PRI-2191 and with or without other anticancer drugs (cisplatin (CIS), docetaxel (DTX) or idarubicin (ID) in the A549 human lung cancer cell line. Moreover, the combined treatment with PRI-2191 or PRI-2205 and imatinib was investigated in mice bearing A549 tumors with the analysis of important molecules targeted by imatinib or vitamin D analogs.

## 2. Results and Discussion

### 2.1. Results

#### 2.1.1. The *in Vitro* Antiproliferative Effect of GV against Human A549 Cells either Combined with PRI-2191 Alone or Applied with Cisplatin, Idarubicin or Docetaxel

Table 1 presents the IC_50_ values (the concentration of the tested compound resulting in a 50% proliferation inhibition of cancer cells) and the combination index (CI) for imatinib (GV) in combination with PRI-2191 and/or docetaxel (DTX), cisplatin (CIS) or idarubicin (ID). The A549 cells were exposed to PRI-2191 (at concentrations of 10 nM or 100 nM) and concurrently to different concentrations of imatinib and/or cytostatic drugs for 96 h. An additive effect in the proliferation inhibition of GV and PRI-2191, examined by a comparison of proliferation plots, was observed (Table 1) [36]. The interactions observed between GV and DTX were antagonistic or additive, but after PRI-2191 at a concentrations of 10 nM was added, these interactions exchanged into synergism in the case of the higher dose of DTX and did not change at the lower dose. PRI-2191 at a concentration of 100 nM transformed the interactions between GV and DTX into synergy. The cooperation of GV with CIS at both doses was additive, but shifted to synergism after incubation with 10 nM or 100 nM of PRI-2191, and the lower dose of CIS. PRI-2191 did not influence the interactions between GV and CIS at a dose of 0.1 μg/mL. By contrast, GV in combination with ID interacted additively (0.001 μg/mL of ID) or antagonistically (at the other dose). PRI-2191 at a dose of 10 nM did not importantly affect the interactions seen in the combination of GV with ID; the additive effect of the triple combination was observed. However, with the use of a 100 nM PRI-2191 concentration, synergism was observed in the triple combination (Table 1).

**Table 1:** The *in vitro* antiproliferative effect against human A549 cells of imatinib mesylate combined with PRI-2191 alone or applied with cisplatin (CIS), idarubicin (ID) or docetaxel (DTX).

Cytostatic (μg/mL)	Dose of PRI-2191 (nM)/Imatinib IC_50_ (μg/mL)					
	100		10		0	
	IC_50_	CI *	IC_50_	CI *	IC_50_	CI *
**	15.71 ± 4.56	Additive *	14.88 ± 3.05	Additive *	22.38 ± 2.07	***
**DTX 0.001**	7.05 ± 3.51	0.629	7.01 ± 2.43	0.627	18.55 ± 8.63	1.266
**0.0001**	16.84 ± 1.4	0.794	20.03 ± 1.65	0.94	19.67 ± 6.01	0.924
**CIS 0.1**	13.77 ± 1.37	0.894	14.91 ± 3.23	0.954	16.28 ± 2.12	1.025
**0.01**	16.86 ± 2.23	0.784	16.2 ± 4.24	0.754	19.9 ± 3.28	0.922
**ID 0.001**	1.42 ± 1.04	0.783	4.24 ± 0.88	0.995	3.08 ± 0.5	0.908
**0.0001**	15.2 ± 2.8	0.793	20.34 ± 2.59	1.038	24.07 ± 2.09	1.216

IC_50_ value for tacalcitol (PRI-2191) alone above 1000 nM; * counted on the basis of proliferation inhibition plots. CI, combination index counted for imatinib mesylate combined with the given cytostatic with or without tacalcitol. A combination index of CI < 0.8 indicated synergism, CI > 1.2 antagonism and CI = 0.8–1.2 an additive effect; ** IC_50_ value for Imatinib without cytostatics; *** CI is countless. The IC_50_ values presented are the mean ± SD obtained for 3–5 separate experiments.

To confirm the profitable *in vitro* effect of vitamin D analogs, we performed *in vivo* experiments using xenotransplantation of a human NSCLC A549 cell line.

#### 2.1.2. The Influence of the Route of PRI-2191 Administration on the Antitumor Effect in Mice Bearing A549 Tumors

**Figure 2:**
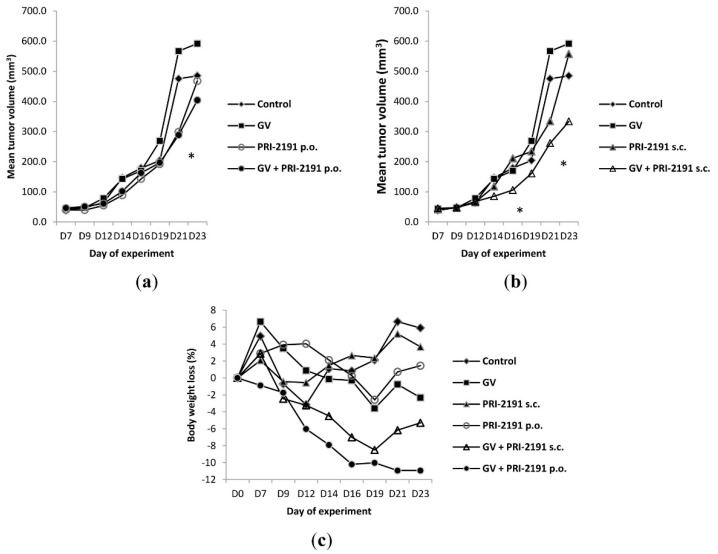
Kinetics of A549 tumor growth after (**a**) oral gavage or (**b**) *s.c.* administration of PRI-2191 analog alone or in combination with Gleevec (GV) and (**c**) body weight changes of treated mice. GV, imatinib; PRI-2191 and PRI-2205, vitamin D analogs; *s.c.*, subcutaneous; (**a**) * *p* < 0.05, Kruskal–Wallis ANOVA, GV + PRI-2191 when compared to imatinib (GV) on Day 21; (**b**) * *p* < 0.05, Kruskal–Wallis ANOVA, GV + PRI-2191 when compared to PRI-2191 on Day 16; GV + PRI-2191 when compared to imatinib (GV) on Day 21. Number of mice per group: control, eight; GV (imatinib), seven; and in each treatment group receiving analog PRI-2191: nine.

The study was performed for the comparison of the anticancer effect revealed by PRI-2191 administered by oral gavage or subcutaneous injections to mice xenotransplanted with A549 tumor and receiving GV treatment. GV injections at a dose of 75 mg/kg/day revealed no inhibition on tumor growth. As shown in Figure 2a,b, the analogue PRI-2191, applied alone or together with GV, revealed different effects on tumor growth depending on the administration course. PRI-2191 alone administered by oral gavage revealed better effect than injected subcutaneously. However, synergistic interactions were observed after subcutaneous injections of PRI-2191 when combined with GV (Figure 2b). When Days 14 and 21 of the experiment were analyzed, both administration schedules show notable tumor growth inhibition regarding the control group or that treated with GV alone. Nevertheless, synergistic interactions between PRI-2191 and GV were observed only after subcutaneous administration. Table 2 presents the tumor volumes and the type of interaction.

**Table 2:** The effect of PRI-2191 alone or in combination with GV on tumor growth in mice bearing subcutaneous human lung cancer A549.

Treatment	Day 14			Day 21		
	Median Tumor Volume (mm^3^)	TGI%	*^H^TGI*	Median Tumor Volume (mm^3^)	TGI%	*^H^TGI*
**vehicle (control)**	154	*	*	460	*	*
**PRI-2191 *s.c.***	98	36	*	305	34	*
**PRI-2191 oral**	92	40	*	263	43	*
**GV**	134	13	*	486	−6	*
**GV + PRI-2191 *s.c.***	76	51	45	262	43	30
**GV + PRI-2191 oral**	96	38	48	287	38	40

TGI, tumor growth inhibition; *^H^TGI*, hypothetical tumor growth inhibition; GV, imatinib; PRI-2191 and PRI-2205, vitamin D analogs; *s.c.*, subcutaneous injection; oral, oral gavage; * countless.

Moreover, independently of the route of PRI-2191 administration, the toxicity of combined treatment was higher than in single-drug therapy (a loss of mice body weight). The highest loss of body weight was noticed in mice with the therapy received orally (but not exceeding 12%) (Figure 2c). This was rather unexpected in light of the previous results that had shown that PRI-2191 given orally revealed lower toxicity than after subcutaneous injections [28].

#### 2.1.3. A Comparison of the Antitumor Activity of PRI-2191 and PRI-2205 in Combined Treatment with GV in Mice Bearing A549 Tumors

**Figure 3:**
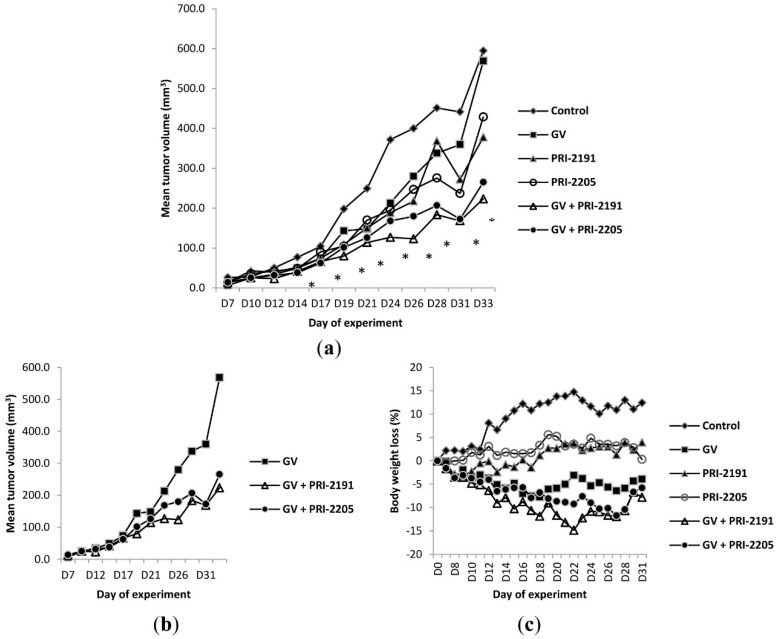
Kinetics of tumor growth in (**a**) all experimental groups and (**b**) groups receiving GV alone or in combination with vitamin D analogs: PRI-2191 and PRI-2205; (**c**) body weight changes of mice bearing A549 tumors, treated with GV and vitamin D analogs: PRI-2191 and PRI-2205. GV, imatinib; PRI-2191 and PRI-2205, vitamin D analogs; (**a**) * *p* < 0.05, Kruskal–Wallis ANOVA, GV + PRI-2191 and GV + PRI-2205 when compared to control group, Days 12–31. Number of mice in groups: control, eight; and in each treatment group, eight.

The aim of this study was to evaluate the activity of PRI-2191 and PRI-2205 injected *s.c.* in a combined treatment with GV in the A549 xenograft model. Lower tumor volume was observed in mice receiving GV alone than in control mice; however, this difference was not statistically significant. A decrease in tumor growth in groups administered both with GV and PRI-2191 or PRI-2205 was also observed. At the 26th day of experiment, a 30% tumor growth retardation was observed in the group treated with GV alone. The combined treatment with GV and analogs PRI-2191 and PRI-2205 resulted in 69% and 55% of tumor growth retardation respectively. As is shown in Figure 3a,b, tumor volume between groups receiving combined treatment and the control group varies significantly for nearly all days of the experiment. Furthermore, the difference in tumor volume for PRI-2191 and GV is also worth mentioning (Figure 3a,b).

Animals treated with GV showed a decrease of mean body weight throughout the whole study. Body weight loss was noticeable in mice from groups that received chemotherapy and the vitamin D analog combined treatment. The body weight decrease of mice treated with both combined GV and PRI-2191 was the highest (15%) on Day 22 of the experiment, but after that day, mice started to recover (Figure 3c).

**Figure 4:**
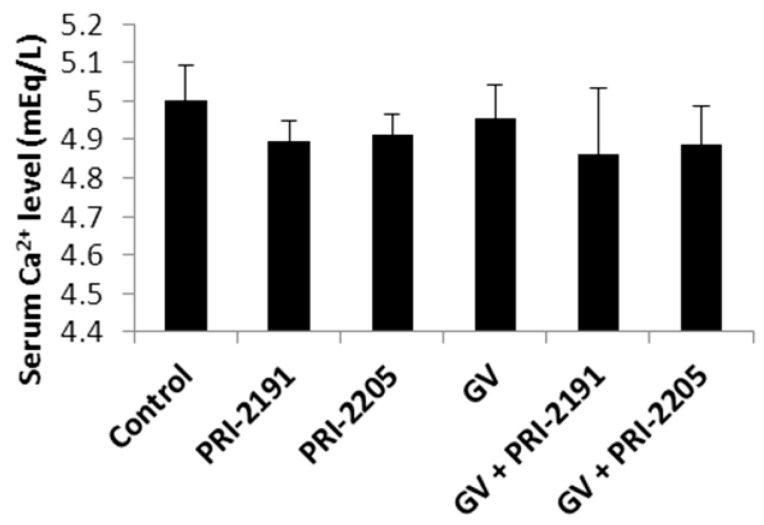
Calcium concentration (mean ± SD) in blood serum collected from mice bearing A549 tumors treated with PRI-2191 and PRI-2205 analogs alone or in combination with GV. GV, imatinib; PRI-2191 and PRI-2205, vitamin D analogs.

The toxicity of calcitriol is related to its concentration; therefore, very high doses of calcitriol have to be used to obtain an anticancer effect, which is then accompanied by elevated serum calcium levels [37,38]. To assess whether vitamin D analogs PRI-2191 and PRI-2205 influenced the calcium concentration, blood from mice was collected, and the level of calcium in serum was measured. As shown in Figure 4, administration of PRI-2191 and PRI-2205 alone or in combination with GV did not affect calcium concentrations in serum.

To assess the mechanism of the observed increased antitumor effect of combined therapy, we analyzed tumors harvested from mice in order to evaluate the influence of such treatment on proteins important in the anticancer activity of both compounds, as well as proteins engaged in vitamin D metabolism.

#### 2.1.4. GV Downregulated the Expression of VEGF in Tumor Tissue Samples

**Figure 5:**
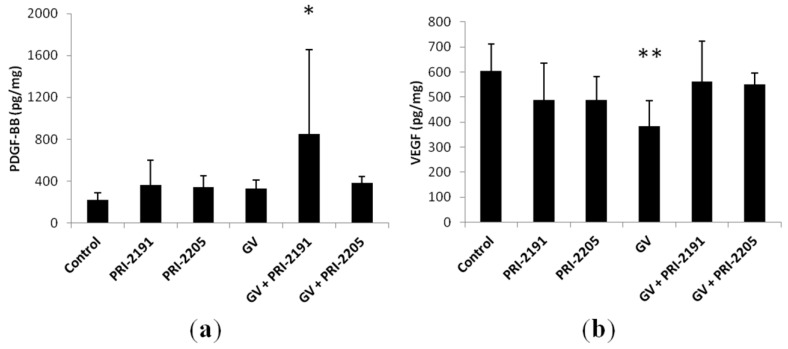
(**a**) PDGF-BB and (**b**) VEGF level (mean ± SD) in A549 tumor lysates harvested from mice treated with PRI-2191 and PRI-2205 alone or in combination with GV. GV, imatinib; PRI-2191 and PRI-2205, vitamin D analogs; * *p* < 0.05 compared to the control and GV, ** *p* < 0.05 compared to the control and GV + PRI-2191, ANOVA followed by Fisher’s test.

The level of VEGF and PDGF-BB in tumor tissue harvested from mice bearing an A549 tumor was analyzed. As shown in Figure 5, treatment with PRI-2191, PRI-2205 and GV alone resulted in downregulation of the VEGF level in A549 tumor tissue compared to the control group. No changes in VEGF level as compared to the control were observed for tumors obtained from mice receiving a combination of GV and both vitamin D analogs. In the case of PDGF-BB, it could be observed that all tested treatment options caused a slight increase in the PDGF-BB level in tumor lysates, and in the case of GV + PRI-2191, PDGF-BB was statistically upregulated compared to control and GV (Figure 5).

**Figure 6:**
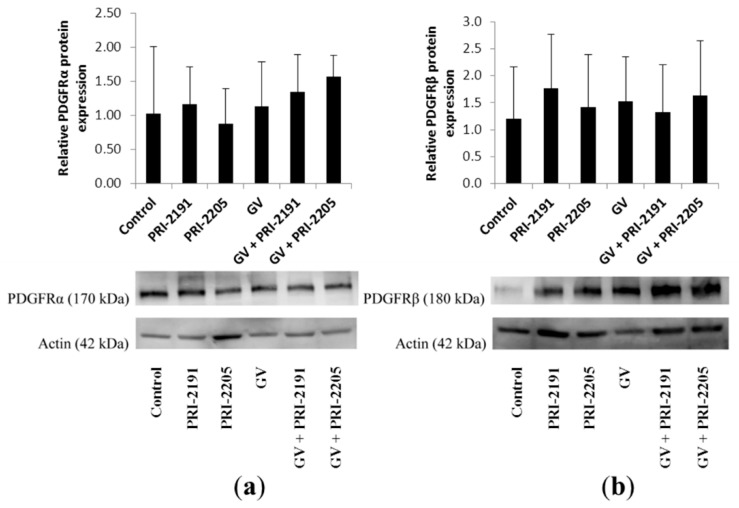
(**a**) PDGFRα and (**b**) PDGFRβ expression in A549 lung cancer tumor tissue determined by Western blot: immunoblot and densitometric analysis (mean ± SD). GV, imatinib; PRI-2191 and PRI-2205, vitamin D analogs.

The expression of PDGF receptors, being the target for imatinib, in tumor tissue harvested from mice bearing an A549 tumor was analyzed. As shown in Figure 6, A549 tumors were positive for PDGFRα and β, but no significant changes in protein expression were observed after the studied treatment.

#### 2.1.5. GV Treatment Upregulated p53 and Downregulated Bcl-2 Expression in A549 Tumors

**Figure 7:**
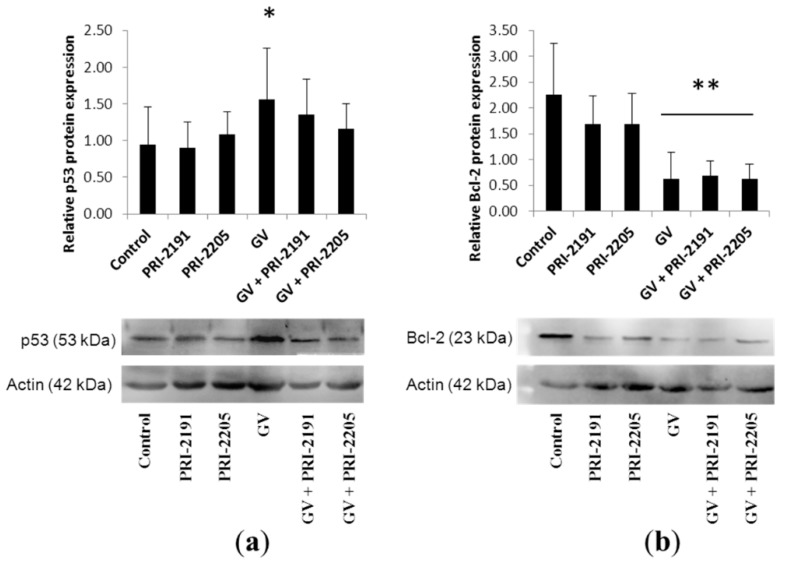
(**a**) p53 and (**b**) Bcl-2 expression in A549 lung cancer tumor tissue determined by Western blot: immunoblot and densitometric analysis (mean ± SD). GV, imatinib; PRI-2191 and PRI-2205, vitamin D analogs; * *p* < 0.05 compared to control and PRI-2191, ** *p* < 0.05 compared to control, PRI-2191 and PRI-2205, ANOVA followed by Fisher’s test.

Western blot analysis of A549 tumor lysates revealed that GV administration resulted in upregulation of p53 tumor suppressor protein expression in the tumor tissue. Neither analog significantly influenced the effect of GV. Moreover, treatment consisting of GV and of GV administered with both analogs resulted in downregulation of Bcl-2 anti-apoptotic proteins in A549 tumors (Figure 7).

**Figure 8:**
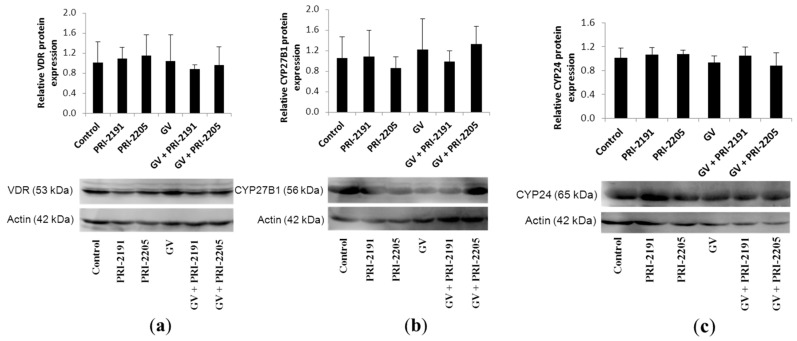
(**a**) VDR, (**b**) CYP27B1 and (**c**) CYP24 expression in A549 lung cancer tumor tissue determined by Western blot: immunoblot and densitometric analysis (mean ± SD). GV, imatinib; PRI-2191 and PRI-2205, vitamin D analogs.

Since other studies have showed a correlation between sensitivity to proliferation inhibition of cultured cells by calcitriol and VDR expression [39], as well as expression of enzymes involved in calcitriol metabolism [40,41], the expression of the following proteins was also analyzed: vitamin D receptor (VDR), CYP27B1 and CYP24. However, no changes in VDR, CYP27B1 and CYP24 were observed between treatment groups (Figure 8).

### 2.2. Discussion

NSCLC is the most common lung cancer and is the main cause of cancer-related deaths worldwide. The development of new agents with improved tolerability profiles has led to curiosity in long-term maintenance therapy in NSCLC patients [42]. It was assumed that inhibition of the macromolecules being targeted for imatinib might augment the effectuality of chemotherapy. For example, PDGFR inhibition should improve the therapeutic effect by decreasing interstitial tumor pressure and, as a result, increased tumor penetration by cytotoxic agents. However, adding imatinib to the treatment with taxanes in metastatic breast cancer, non-small cell lung cancer or head and neck cancer patients in phase II clinical trials showed that such therapy was comparatively poorly tolerated and had low objective response rates [43,44,45,46].

Encouraged by the possibility to improve the anticancer activity of imatinib by vitamin D analogs, including in triple combination with a cytostatic drug, we conducted a series of *in vitro* and *in vivo* experiments aimed at obtaining a beneficial anticancer effect from combined treatment without additional side effects.

In our previous studies, PRI-2191 enhanced the antiproliferative effect of imatinib alone or with both docetaxel and cisplatin, but not with idarubicin on HL-60 cells. What is important is that PRI-2191 could shift the antagonistic or additive interaction between the chemotherapy agents to synergism [9]. As we showed in that paper, the same observation was made in the case of A549 lung cancer cells. Vitamin D analog PRI-2191 augmented the antiproliferative activity of GV used in combination with tested chemotherapy agents against A549 cells.

In order to confirm our observation from *in vitro* studies with GV and vitamin D analog PRI-2191 on A549 cells, we designed preliminary *in vivo* experiment with mice bearing A549 *s.c.* tumors in order to assess the influence of the route of vitamin D analog administration on GV anticancer activity. As we showed in this paper, subcutaneous injection of vitamin D analog PRI-2191 in combination with GV revealed better activity and lower toxicity. Therefore, the experiment with the use of another vitamin D analog, PRI-2205, characterized as having extremely low toxicity [15], was carried out using the subcutaneous route of administration. Moreover, we also reconsidered the dose of PRI-2191 on the basis of our results showing that a dose of 1 μg/kg/day of PRI-2191 is the best balance between activity and toxicity in mice bearing murine MC-38 colon cancer [32]. Our study showed that coadministration of GV together with vitamin D analogs PRI-2191 or PRI-2205 revealed stronger antitumor activity in the *in vivo* A549 lung cancer model. In our previous studies, it was shown that vitamin D analogs augmented anticancer activity of cisplatin in the LLC (Lewis lung cancer) model [17], and in Zhuravel *et al.*’s study, vitamin D_3_ elevated the antimetastatic effect of protein-based vaccine containing murine β-defensin-2 (mBD-2) and Lewis lung cancer 3LL cell lysate in the same model [47]. However, it had been demonstrated that combined treatment with CIS and vitamin D analogs in mice bearing Lewis lung cancer caused toxicity manifested in body weight loss, leukopenia and hypercalcemia. Interestingly, the toxicity of this treatment protocol was not diminished by clodronate bisphosphonate [17]. In the present study with the A549 lung cancer model, the toxicity observed during the treatment schedule was probably not dependent on serum calcium level, as observed in our previous results.

A549 cells are known to lack expression of PDGFR α and β, one of the targets for imatinib, but in lung cancer, these receptors are frequently expressed by tumor-associated stromal cells [48,49]. Involvement of PDGFR α in the recruitment of stromal fibroblasts producing VEGF for tumor growth and angiogenesis was described, whilst PDGFR β has an important role in angiogenesis, since it is required for pericyte recruitment, which contributes to vessel maturation and stabilization and modulates vascular permeability. Thus, targeting the PDGF-PDGFR axis, even when malignant cancer cells do not express these molecular targets for imatinib, can be a promising treatment strategy [48]. In our experiments, we could observe an increase in PDGF-BB expression in tumor tissue after applied treatment. Abe *et al.* showed that imatinib treatment in a pancreatic cancer model resulted in upregulation of human PDGF-B expression, with simultaneous downregulation of mouse PDGF-B production. It could be concluded that cancer cells, in order to compensate the inhibition of the PDGF-B/PDGFR β axis, started to produce higher levels of PDGF-B [50]. Moreover, calcitriol is known to be an agent that diminishes the expression of PDGFR during HL-60 cell differentiation [51]. On the other hand, calcitriol exerts profound antiproliferative effects on TDEC (tumor-derived endothelial cells) by forcing G_0_/G_1_ cell cycle arrest and apoptosis, affecting the development of tumor angiogenesis *in vivo*. Treatment of TDEC with calcitriol resulted in increased VDR protein expression and activation of the VDR signaling pathway. What is more, highly aberrant vasculature is observed in tumor blood vessels in VDR knock-out (KO) mice. Compared to wild-type animals, the tumor vessels in KO mice are enlarged and are associated with fewer pericytes, and the tumors contain increased contents of angiogenic factors, such as HIF-1α, VEGF, Ang1 and PDGF-BB [20]. In our study, tumors harvested from mice bearing A549 xenografts were positive for PDGFR α and β, but no significant changes in PDGFR α or β expression were observed after the studied treatment. However, our recent studies on HLMEC cells (data not published yet) show that calcitriol and vitamin D analogs increase the cytotoxic effect towards these cells of anticancer agents *in vitro*. Therefore, vitamin D analogs could influence the process of angiogenesis in tumor tissue by forcing apoptosis of endothelial cells.

Calcitriol or its analogs reveal various activities, balanced between pro- and anti-apoptotic ones. Particularly, calcitriol upregulated the level of the pro-apoptotic BAK protein (member of the BCL-2 family) [52] and decreased the anti-apoptotic activity of β-catenin [53]. Furthermore, it was shown that p21^waf1/cip1^ (one of the target genes of p53) is a gene primarily responding to calcitriol; it contains VDR binding promoter regions, in which p53 also co-localizes [54]. In our previous *in vitro* studies using HT-29 colon cancer cells, antagonism in proapoptotic activity, mainly of PRI-2191 and 5-fluorouracil (5-FU), was suggested. The percentage of propidium iodide-stained cells, p53-positive cells, as well as the activity of caspase-3 decreases compared to 5-FU alone. On the other hand, treatment with HT-29 cells with PRI-2205 led to a slight decrease only of propidium iodide-stained cells, but active caspase-3 or p53 expression as compared to 5-FU alone was not affected. HT-29 cells expressed mutant p53 [55]; therefore, interaction between that mutant p53 and the vitamin D pathways could lead to an anti-apoptotic state [56]. Nevertheless, regardless of these disadvantageous effects concerning apoptosis, the therapeutic effect in the form of colon tumor growth retardation was significant. In contrast, PRI-2205, several activities of which differ from those observed for calcitriol or PRI-2191 (e.g., lack of influence on the expression of p53), did not show anti-apoptotic activity in a colon cancer model [57]. Moreover, our previous studies, for example with HL-60 leukemia cell lines, revealed the pro-apoptotic activity of this analog [15].

There are many studies concerning drugging the p53 pathway, since it is implicated in a growing number of cellular processes: cell cycle arrest, DNA repair, apoptosis, autophagy, metabolism and gene transcription regulation, whose dysregulation is engaged in cancerogenesis [58]. Therefore, treatment strategies targeting upregulation of the p53 pathway involved in so many biological events (as listed above), especially for those cancers with wild-type p53, may offer extremely positive outcomes. It is also already known that p53 is involved in regulating new blood vessel formation, and uncontrolled angiogenesis is associated with the progression of cancers. The inhibition of angiogenesis by p53 is mediated through involvement in the hypoxia regulatory machinery, inhibition of the production of proangiogenic factors and upregulation of the production of angiogenesis inhibitors [59]. As shown in this study, treatment of A549 tumors with GV resulted in upregulation of p53 expression with simultaneous downregulation of VEGF in tumor tissue.

Recent studies have shown that treatment with imatinib modulates the expression of the Bcl-2 family of apoptosis regulators with favorable proapoptotic phenotypes [60,61]. Moreover, our results also showed that treatment of mice bearing A549 tumors either with imatinib alone or in combination with the tested analogs resulted in downregulation of Bcl-2 proteins in A549 tumor tissue. The Bcl-2 protein belongs to a family of proteins controlling the intrinsic pathway of apoptosis, and Bcl-2 itself acts as a prosurvival protein preventing cells from apoptosis. Bcl-2 is often overexpressed in many cancers, including leukemia, lymphoma, melanoma, non-small lung cancer, prostate, breast, gastric and colon carcinoma. Moreover, overexpression of Bcl-2 is believed to be involved in inducing drug resistance to chemotherapy [62].

Therefore, the observed tumor growth inhibition after treatment with imatinib and vitamin D analogs, PRI-2191 and PRI-2205, could be the result of apoptotic cell death, since downregulation of anti-apoptotic Bcl-2 protein was observed. Moreover, the administration of imatinib can modulate angiogenesis in tumors by lowering the level of VEGF, probably due to upregulation of p53. However, the exact mechanism of vitamin D analog contribution to the anticancer activity of imatinib needs to be further elucidated.

## 3. Experimental Section

### 3.1. Cell Culture

The *in vitro* cultured human lung cancer A549 cell line was obtained from the American Type Culture Collection (ATCC, Bethesda, MA, USA). The cells were maintained in RPMI-1640 GlutaMAX (Gibco, Paisley, UK) medium with the addition of 1.0 mM sodium pyruvate, 4.5 g/L glucose (both from Sigma-Aldrich Chemie GmbH, Steinheim, Germany), 100 U/mL penicillin, 100 μg/mL streptomycin (both from Polfa Tarchomin S.A., Warsaw, Poland) and supplemented with 10% fetal bovine serum (Sigma-Aldrich Chemie GmbH, Steinheim, Germany). The cells were maintained under standard cell culture conditions (humidified atmosphere and 5% CO_2_ at 37 °C).

### 3.2. Compounds

The calcitriol analogs, (24*R*)-1,24-dihydroxyvitamin D_3_ (1,24(OH)_2_D_3_, PRI-2191, tacalcitol) and 5,6-trans calcipotriol (PRI-2205) (Figure 1), were provided by the Pharmaceutical Research Institute (Warsaw, Poland). Compounds were stored in amber ampoules under argon at −20 °C. Prior to use in *in vitro* tests, the agents were dissolved in 99.8% ethanol to a concentration of 0.1 mM and next diluted in culture medium to reach the required concentrations. Tested anticancer drugs were cisplatin (CIS), obtained from KZF Polfa (Krakow, Poland), docetaxel (DTX) from Sigma-Aldrich Chemie GmbH (Steinheim, Germany), imatinib mesylate (GV) and idarubicin (ID) from the Pharmaceutical Research Institute (Warsaw, Poland).

Prior to *in vivo* usage, PRI-2191 or PRI-2205 were dissolved in 99.8% ethanol, next diluted in 80% propylene glycol to reach the desirable concentrations and administered to mice at a volume of 5 μL/g of body weight. Imatinib mesylate (GV) was diluted in water for injectionto reach the required concentrations and administered to mice at a volume of 10 μL/g of body weight.

### 3.3. In Vitro Anti-Proliferative Assay

Tested A549 cells were placed in 96-well flat-bottom plates (Sarstedt, Inc., Newton, NC, USA) at a density of 5 × 10^3^ cells per well 24 h before the addition of the test compounds. The cells were incubated for 96 h with two different concentrations (10 and 100 nM) of PRI-2191 and concurrently with various concentrations of GV (10, 100, 1000 and 10,000 ng/mL) and other cytostatic drugs (DTX or ID: 0.1, 1, 10, 100 ng/mL; CIS: 1, 10, 100, 1000 ng/mL). The sulforhodamine B (SRB) assay was performed to evaluate the cytotoxic effect, as described previously [15]. As a result, IC_50_ (inhibitory concentration 50%), *i.e.*, the dose of the tested compound that inhibited the proliferation of cancer cells by 50%, was calculated for each separate experiment in Cheburator 0.4, Dmitry Nevozhay software [63]. The mean values ± SD are presented in Table 1. Each compound concentration in a given experiment was tested in triplicate; each experiment was repeated 3–5 times. Ethanol, serving as the solvent of the tested agents, in a dilution corresponding to its highest concentration applied with the tested compounds, did not exert any inhibitory effect on cell proliferation (*p* > 0.05).

#### Analysis of Combination Effects

The cytotoxic activity obtained for the different combinations of the vitamin D analog with the cytostatic drugs was analyzed according to the method of Chou and Talalay [64,65]. The interaction between two compounds (mutually non-exclusive) was estimated using the combination index (CI) calculated for the IC_50_s from the *in vitro* experiments:

**CI_A+B_** = (**D_A/A+B_**/**D_A_**) + (**D_B/A+B_**/**D_B_**) + (**D_A/A+B_** × **D_B/A+B_**)/**D_A_D_B_** (1)

where **CI_A+B_** is the combination index for the experimentally achieved effect F (IC_50_) for the combination of Compound A (the given cytostatic) and Compound B (calcitriol or analog), **D_A/A+B_** the concentration of Compound A in the combination A + B giving the effect F, **D_B/A+B_** the concentration of Compound B in the combination A + B giving the effect F, **D_A_** the concentration of Compound A alone giving the effect F and **D_B_** the concentration of Compound B alone giving the effect F.

A combination index of CI < 0.8 indicated synergism, CI > 1.2 antagonism, and CI = 0.8–1.2 an additive effect.

### 3.4. Animals

NOD/SCID female mice, 12–16 weeks old, body weight of 20–25 g, supplied from the Nofer Institute of Occupational Medicine (Lodz, Poland), were maintained in specific pathogen free SPF conditions. Mice were fed with standard Ssniff^®^ R/M-H (Ssniff Spezialdiäten GmbH, Soest, Germany) diet with 1000 IU/kg of vitamin D. All experiments were carried out in accordance with the national and international rules concerning the Care and Use of Laboratory Animals including EU Directive 2010/63/EU for animal experiments and were approved by the 1st Local Committee for Experiments with the Use of Laboratory Animals, Wroclaw, Poland (No. 42/2005, accepted: 14 December 2005).

### 3.5. Design of the in Vivo Experiments

Mice were subcutaneously (*s.c.*) inoculated in the right flank of the abdomen with A549 tumor cells suspension (5 × 10^6^ cells in 0.2 mL of Hank’s medium per mouse (Day 0)) and then were randomized into groups receiving varied combinations of vitamin D analogs and chemotherapeutics. One out of two experimental protocols was applied in the respective experiments:

1. The treatment was started from Day 7 after inoculation of tumor cells (when tumors became palpable). GV was administered intraperitoneally (*i.p.*) at a dose of 75 mg/kg/day, daily for 19 days (from Days 7–25). PRI-2191 was administered *s.c.* or by oral gavage at a dose of 2 μg/kg/day, 3 times a week (on Days 7, 12, 14, 16, 19, 21 and 23).
2. The treatment was started from Day 7 after inoculation of tumor cells (when tumors became palpable). GV was administered intraperitoneally (*i.p.*) at a dose of 50 mg/kg/day, daily for 13 days (from Days 7–19). PRI-2191 and PRI-2205 were administered *s.c.* at doses of 1 or 10 μg/kg/day, respectively, 3 times a week (on Days 7, 10, 12, 14, 17, 19, 21, 24 and 26).

At the end of the experiments, blood was collected under anesthesia; then, the mice were sacrificed.

#### Evaluation of the Therapeutic Effect

The following formula was used for tumor volume calculation (a^2^ × b)/2, where a = shorter tumor diameter in mm and b = longer tumor diameter in mm. Inhibition of tumor growth was calculated from the following formula: TGI (%) (tumor growth inhibition) = (VT/VC) × 100 − 100%, where VT is the median tumor volume of treated mice and VC the median tumor volume of control animals (receiving no treatment).

### 3.6. Calcemic Activity

Animals were sacrificed at the end of the experiment, and blood sera of all animals were collected. The calcium level was measured in each individual serum sample by means of the photometric Arsezano 3 method (Olympus AU400; Olympus America Inc., Melville, NY, USA).

### 3.7. Western Blot Analysis

Tumor tissues harvested from mice treated with imatinib and/or vitamin D analogs were frozen in liquid nitrogen, and after storage at −80 °C, tissues were homogenized (Tissue and Cell Homogenizer FastPrep-24, MP Biomedicals, Warsaw, Poland) in RIPA buffer supplemented with a protease inhibitor cocktail (Sigma-Aldrich Chemie GmbH, Steinheim, Germany). The total protein concentration in the obtained tissue lysates was measured with the Bradford assay (Bio-Rad, Warsaw, Poland). One hundred micrograms of protein were denatured and loaded onto SDS-PAGE gels and, after electrophoresis, transferred to PVDF membranes (GE Healthcare UK Limited, Little Chalfont, UK). After blocking in 5% non-fat dry milk in PBS, membranes were probed with the following primary antibodies: anti-p53, anti-Bcl-2, anti-VDR, anti-CYP24, anti-CYP27B1, anti-PDGFRα, anti-PDGFRβ and anti-actin (Santa Cruz Biotechnology, Santa Cruz, CA, USA), followed by alkaline phosphatase conjugated secondary antibody; and signals were obtained with an enhanced chemifluorescence kit (GE Healthcare UK Limited, Little Chalfont, UK). Images were acquired with a Carestream Image Station 4000 MM Pro (Carestream Health, Inc., New Brunswick, NJ, USA). Western blot analysis was repeated in triplicate. Densitometric analysis of the Western blots was performed in ImageJ 1.48 v software (National Institutes of Health, Bethesda, MA, USA). Blots were normalized to actin. Western blot analysis was repeated for three to four tumor lysates of all treatment groups.

### 3.8. PDGF-BB and VEGF ELISA Assay

Levels of PDGF-BB and VEGF in tumor lysates were assessed using commercially-available ELISAs kits (eBiosciences, Vienna, Austria and Invitrogen, Camarillo, CA, USA respectively), following the manufacturer’s instructions. Tumor lysates were prepared as described above. The cytokine level was next normalized in each sample to the total protein concentration. The cytokine level was obtained for four tumor lysates in each experimental group.

### 3.9. Statistical Evaluation

Statistical analysis was performed by means of STATISTICA Version 7.1 software (StatSoft, Inc., Tulsa, OK, USA). The Kruskal–Wallis ANOVA multiple comparison *p*-value (two-tailed) test and the Mann–Whitney U-test were used for tumor growth inhibition analysis. *p*-values less than 0.05 were considered as significant.

## 4. Conclusions

In our study, we have demonstrated that vitamin D analogs augment the anticancer activity of imatinib in an NSCLC model. The use of PRI-2191 modulates the interaction of imatinib with standard cytostatic drugs in the A549 NSCLC model *in vitro*. When the interaction between imatinib and cytostatic drug were antagonistic or additive, the addition of PRI-2191 shifted it to synergism.

The antitumor effect *in vivo* of PRI-2191 alone or in combined treatment with GV depends on its administration route. Oral administration of PRI-2191 used alone exhibits better activity than after *s.c.* injections. Nevertheless, in combined treatment with GV, synergistic cooperation could be observed when PRI-2191 was given subcutaneously. Independent of the route of PRI-2191 administration, the toxicity of the combined treatment was higher than in single-drug therapy (a decrease of the body weight of mice). Administration of PRI-2191 and PRI-2205 alone or in combination with GV did not affect calcium concentration in serum.

The observed tumor growth inhibition after treatment with imatinib and vitamin D analogs, PRI-2191 and PRI-2205, could be the result of apoptotic cell death, since downregulation of anti-apoptotic Bcl-2 protein was observed. Treatment with PRI-2191, PRI-2205 and GV alone, but not in combination, resulted in downregulation of the VEGF level in A549 tumor tissue. Therefore, the administration of imatinib can modulate angiogenesis in tumors by lowering the level of VEGF, probably due to upregulation of p53. The exact mechanism of the vitamin D analog contribution to anticancer activity of imatinib needs to be further elucidated.
